# Harnessing the (CH_3_)_2_ZnCl^–^ Anion for Dimethylzinc Stabilization as a Pathway to Stable Dimethylzinc Salts and Dimethylzinc Recovery

**DOI:** 10.1021/acs.jpca.5c00568

**Published:** 2025-03-19

**Authors:** Dawid Falkowski, Alicja Mikolajczyk, Piotr Skurski

**Affiliations:** † Faculty of Chemistry, University of Gdańsk, Wita Stwosza 63, Gdańsk 80-308, Poland; ‡ QSAR Lab Ltd., Trzy Lipy 3, Gdańsk 80-172, Poland; § Department of Chemistry, University of Utah, Salt Lake City, Utah 84112, United States

## Abstract

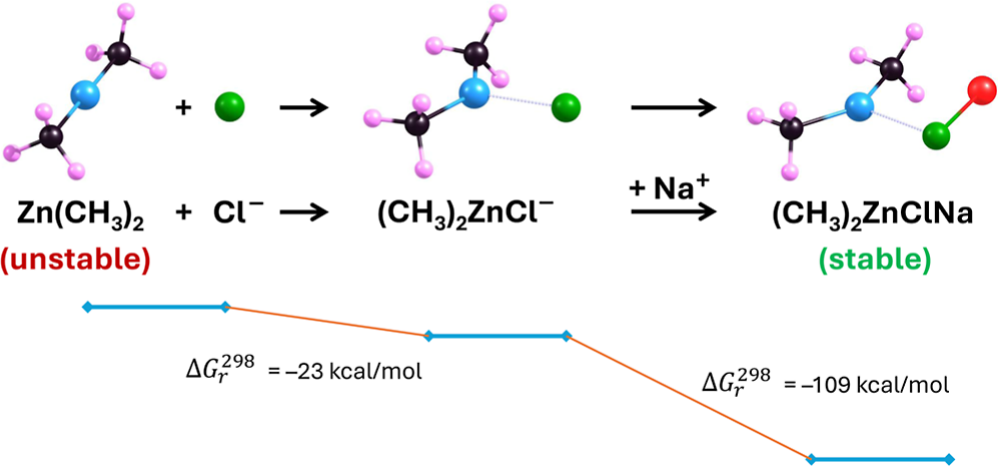

The possibility of stabilizing reactive dimethylzinc through salt formation has been investigated using advanced *ab initio* electronic structure methods and flexible basis sets. It was found that the attachment of a Cl^–^ ion to dimethylzinc is thermodynamically favorable (with a Gibbs free reaction energy of −22.88 kcal/mol at room temperature), occurring without a kinetic barrier. The resulting anion is strongly electronically bound, with an excess electron binding energy of 4.306 eV. The subsequent attachment of Li^+^ or Na^+^ ions to this anion leads to the formation of ionic salts (CH_3_)_2_ZnClLi or (CH_3_)_2_ZnClNa. These salts, formed through this two-step process, are thermodynamically stable and represent stabilized forms of dimethylzinc, from which the pure dimethylzinc compound can be regenerated via the procedures suggested in this work. In addition to the structural characterization of these systems and a detailed analysis of the electronic structure of the (CH_3_)_2_ZnCl^–^ anion, which plays a key role in the described process, experimental approaches for realizing each transformation are also proposed.

## 1 Introduction

Dimethylzinc (Zn(CH_3_)_2_) is a highly reactive organozinc compound^1,2^ widely used in organic synthesis, catalysis, and materials science.^3^ As a colorless, volatile liquid at room temperature, it is characterized by its pyrophoric nature, igniting spontaneously upon exposure to air. The high reactivity of dimethylzinc stems from its polarized zinc–carbon bonds, where the zinc center acts as an electrophile and the methyl groups as nucleophiles. These properties make dimethylzinc a versatile reagent, though its extreme sensitivity to oxygen and moisture presents significant handling challenges, requiring an inert atmosphere for safe use.^4,5^

One of the primary applications of dimethylzinc is in organic synthesis, where it serves as an effective methylation agent. It readily transfers methyl groups to various electrophilic substrates, such as carbonyl compounds and alkyl halides, making it a valuable reagent for carbon–carbon bond formation. In particular, dimethylzinc has been employed in the synthesis of complex organic molecules, including pharmaceuticals and fine chemicals. Additionally, its use in catalytic processes, such as in the formation of Grignard-type reagents, further emphasizes its importance in modern organic chemistry.^6−10^

Dimethylzinc also plays a critical role in the semiconductor industry, particularly in the process of metalorganic chemical vapor deposition (MOCVD).^11−14^ In this process, dimethylzinc serves as a precursor for the deposition of high-purity zinc oxide (ZnO) and other zinc-based materials. These materials are essential in the fabrication of optoelectronic devices such as light-emitting diodes (LEDs), laser diodes, and solar cells. The precise control over thin film growth provided by MOCVD makes dimethylzinc indispensable in the production of advanced semiconductor materials used in electronics and photonics.^15^ Moreover, the tunable properties of the resulting zinc oxide films, including their conductivity and optical characteristics, have sparked interest in developing new materials for next-generation electronic devices.^16,17^ The versatility and effectiveness of dimethylzinc in these applications highlight its importance in the ongoing advancement of semiconductor technologies.^18^

Despite its wide range of applications, the handling of dimethylzinc presents significant challenges due to its extreme sensitivity to moisture and air. Upon exposure to oxygen, dimethylzinc ignites spontaneously, while contact with water results in the rapid formation of zinc hydroxide and methane gas. These reactions not only pose safety hazards but also complicate the use of dimethylzinc in laboratory and industrial settings, necessitating strict protocols for storage and handling under inert conditions. The importance of understanding these safety considerations cannot be overstated, as improper handling can lead to severe accidents and environmental hazards.^19^

The reactivity of dimethylzinc is not limited to its applications and handling challenges; it also extends to its ability to form complexes with various ligands.^20^ Coordination interactions between dimethylzinc and oxygen- or nitrogen-based ligands can significantly modify its stability and reactivity. These interactions can help mitigate the inherent reactivity of dimethylzinc, enhancing its stability and allowing for safer handling.^21−25^

In this work, we explore novel pathways for stabilizing dimethylzinc through the attachment of the chloride anion, followed by its neutralization with alkali metal ions, leading to the formation of thermodynamically stable salts. We also suggest experimental approaches to generating these stable salts of dimethylzinc, which could broaden its applicability in diverse chemical processes and applications. Since an analogous approach, in a certain sense, was applied by Knight et al. to stabilize highly pyrophoric Al(BH_4_)_3_ (by first converting Al(BH_4_)_3_ to Al(BH_4_)_4_ and then to a stable KAl(BH_4_)_4_ salt),^26^ we believe that the procedure described in our work may similarly prove effective in stabilizing the highly reactive compound Zn(CH_3_)_2_.

## 2 Methods

The equilibrium structures and the corresponding harmonic vibrational frequencies of the isolated neutral dimethylzinc molecule (Zn(CH_3_)_2_), the (CH_3_)_2_ZnCl^–^ anion, the (CH_3_)_2_ZnClLi and (CH_3_)_2_ZnClNa salts, and their fragmentation products were determined using the quadratic configuration interaction method with single and double substitutions (QCISD)^27−29^ with the aug-cc-pVDZ basis set.^30^ We examined the lowest eigenvalue of the atomic orbital overlap matrix to confirm that near-linear dependency was not an issue.

To verify whether the theoretical treatment applied (i.e., QCISD/aug-cc-pVDZ) yields reliable bond lengths, valence, and dihedral angles, we determined the stationary point structures for two selected systems, namely, the neutral Zn(CH_3_)_2_ molecule and the (CH_3_)_2_ZnCl^–^ anion, using (i) the same QCISD method with a larger aug-cc-pVTZ basis set,^31^ and (ii) the coupled cluster method with single and double substitutions (CCSD)^32−35^ together with the aug-cc-pVDZ basis set. Since the bond lengths and valence and dihedral angles predicted at the QCISD/aug-cc-pVTZ level differed only slightly from the corresponding values obtained at the QCISD/aug-cc-pVDZ level (with maximum differences not exceeding 0.01 Å for bond lengths and 0.3° for angles), and the values obtained at the CCSD/aug-cc-pVDZ level also differed minimally from those determined at the QCISD/aug-cc-pVDZ level (with maximum differences not exceeding 0.005 Å for bond lengths and 0.1° for angles), we are confident that the QCISD/aug-cc-pVDZ approach used for the structural determination of the systems investigated in this work is sufficiently accurate.

The relaxed scan of the potential energy surface of (CH_3_)_2_ZnCl^–^ (i.e., partial geometry optimizations assuming a certain interatomic separation (i.e., Zn–Cl distance) frozen and the other bond lengths and angles relaxed to minimize the energy) was performed at the same QCISD/aug-cc-pVDZ theory level.

The vertical electron detachment energies (VDE) characterizing the (CH_3_)_2_ZnCl^–^ anion were calculated by applying the outer valence Green function OVGF method (*B* approximation)^36−44^ together with the aug-cc-pVDZ,^29^ aug-cc-pVTZ,^30^ aug-cc-pVQZ,^45^ and aug-cc-pV5Z^46^ basis sets to achieve basis set saturated result. Therefore, we are confident that increasing the basis set further would not significantly affect the predicted VDE value. Due to the fact that the OVGF approximation remains valid only for outer valence ionization for which the pole strengths (PS) are greater than 0.80–0.85,^47^ we verified that the PS values obtained were sufficiently large (i.e., spanning the 0.899–0.901 range) to justify the use of the OVGF method.

The Gibbs free reaction energies at *T* = 298.15 K (Δ*G*_r_^298^) for the formation of the (CH_3_)_2_ZnCl^–^ anion and the (CH_3_)_2_ZnClM (M = Li, Na) salts, as well as for their fragmentation processes, were calculated using the electronic energies, zero-point energy corrections, thermal corrections and entropy contributions, all estimated at the QCISD/aug-cc-pVDZ theory level.

The partial atomic charges and bond occupancies were evaluated (using QCISD/aug-cc-pVDZ electron densities) by the Natural Bond Orbital (NBO) analysis scheme^48−52^ employing the NBO 7.0 software.^53^

All calculations were performed with the GAUSSIAN16 (Rev.C.01) package.^54^

## 3 Results and Discussion

In this work, we aim to present an alternative approach to stabilizing dimethylzinc, a compound known for its high reactivity and strong reducing properties, which is typically sold as a solution in alkanes (e.g., hexane) or toluene. Instead, we propose the possibility of stabilizing dimethylzinc through the formation of salts (CH_3_)_2_ZnLiCl or (CH_3_)_2_ZnNaCl.

We clarify here that in the context of organozinc chemistry, compounds like (CH_3_)_2_ZnClLi and (CH_3_)_2_ZnClNa can indeed be referred to as salts because they are formed from the coordination of the zinc-containing anion (CH_3_)_2_ZnCl^–^ with lithium or sodium cations. Since salts are generally defined as ionic compounds resulting from the interaction between ions, the (CH_3_)_2_ZnCl^–^ anion acts as a Lewis base, donating electron density, while the lithium or sodium cations, being electron-deficient, act as Lewis acids by accepting electron density, thus resulting in the formation of an ionic compound. In fact, many organozinc compounds can be classified as salts, particularly those that include a metal cation paired with a negatively charged organozinc anion. The (CH_3_)_2_ZnLiCl and (CH_3_)_2_ZnNaCl compounds under consideration fit this classification and represent a unique form of organozinc salts due to their ionic nature and the coordination of cations.

Our proposed approach to stabilizing dimethylzinc involves a two-step process, with the first step being the attachment of a Cl^–^ anion to the Zn(CH_3_)_2_ molecule, forming the (CH_3_)_2_ZnCl^–^ anion, and the second step being the neutralization of this anion with a Li^+^ or Na^+^ cation. As we will demonstrate in this section, both of these processes are thermodynamically favorable and occur without kinetic barriers.

In discussing the detailed results of our *ab initio* theoretical calculations for both stages leading to the formation of the salts (CH_3_)_2_ZnClLi and (CH_3_)_2_ZnClNa, as well as the process of regenerating dimethylzinc from these salts, we also provide suggestions for implementing these processes experimentally. However, we emphasize that the experimental recommendations provided here should be regarded strictly as suggestions, as we fully recognize that these cannot be verified through theoretical calculations alone.

### 3.1 Justification for the Two-step Approach

To begin with, we would like to explain why, in the approach we propose, we adopted a two-step process: first attaching a chloride anion to dimethylzinc to form (CH_3_)_2_ZnCl^–^, followed by neutralizing (CH_3_)_2_ZnCl^–^ with a Li^+^ or Na^+^ cation to form the salts (CH_3_)_2_ZnClLi or (CH_3_)_2_ZnClNa, rather than employing a one-step process involving the direct reaction of dimethylzinc with LiCl or NaCl.

First, the two-step approach allows for better control over anion formation, as this targeted strategy ensures that the halide ion coordinates effectively to the dimethylzinc molecule, forming the desired anionic species. In contrast, a direct reaction with LiCl or NaCl would involve simultaneous competition between Li^+^/Na^+^ and Cl^–^ for coordination with dimethylzinc. This could result in inefficient or incomplete halide coordination, where Li^+^ or Na^+^ interferes, producing a less stable or mixed product (along with the risk of Li^+^ or Na^+^ forming various salt or complex structures, resulting in products with inconsistent composition). By separating the stages, this competition can be avoided.

Second, directly reacting dimethylzinc with LiCl or NaCl introduces the risk of immediate ion pairing between Zn(CH_3_)_2_ and Li^+^/Na^+^, which could destabilize the reaction environment. In such a scenario, Li^+^ or Na^+^ might prematurely coordinate with Zn(CH_3_)_2_ before Cl^–^ has fully attached, leading to less defined products or incomplete complexation. This premature interaction could produce a mixture of species, including uncoordinated or partially coordinated dimethylzinc, reducing the yield and complicating purification. By first forming the (CH_3_)_2_ZnCl^–^ anion and then adding Li^+^ or Na^+^ in a controlled second step, one can ensure a clean and stable formation of the desired salt.

Third, the two-step procedure allows one to avoid solubility and mixing issues. Dimethylzinc and LiCl or NaCl have different solubility properties, which could lead to poor mixing and inefficient reactions when combined directly. LiCl and NaCl are highly ionic salts, generally insoluble in the nonpolar solvents typically used for organozinc compounds. As a result, a direct reaction may proceed poorly due to inadequate contact between the reactants. By first introducing a halide source that coordinates more effectively with dimethylzinc in the chosen solvent system, and then neutralizing the anionic complex with Li^+^ or Na^+^, it is possible to optimize the conditions for a smooth and complete reaction, avoiding solubility limitations.

Lastly, there are kinetic considerations and reaction efficiency to address. A direct reaction between dimethylzinc and LiCl or NaCl could suffer from unfavorable kinetics due to the simultaneous introduction of both a cation and an anion. The presence of Li^+^ or Na^+^ could alter the pathway of halide coordination, slowing down or even hindering the formation of the (CH_3_)_2_ZnCl^–^ anion. This could result in a slow reaction or incomplete product formation. By separating the steps, the halide coordination occurs first under optimal conditions, allowing for faster and more efficient reaction kinetics when Li^+^ or Na^+^ is introduced in the second stage.

### 3.2 Formation of the (CH_3_)_2_ZnCl^–^ Anion

**Figure 1:**
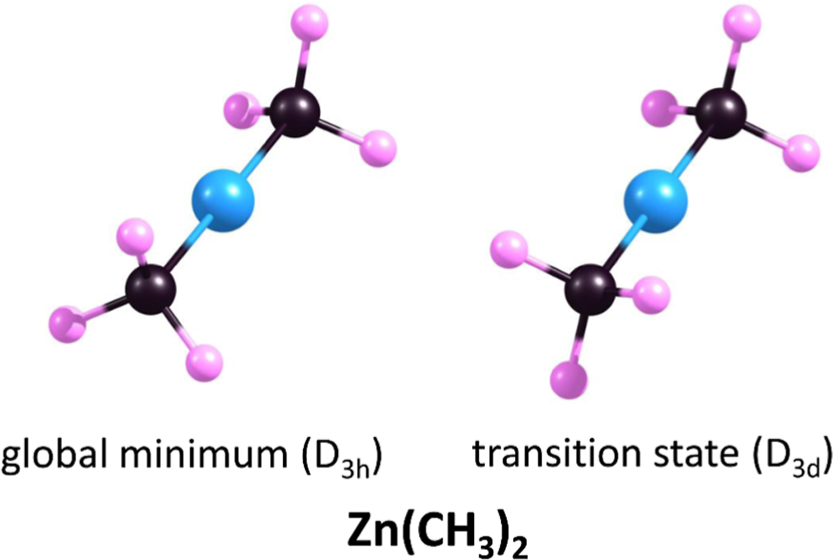
Equilibrium structures of the global minimum and transition state of neutral dimethylzinc molecule.

Our calculations revealed that the lowest energy structure of Zn(CH_3_)_2_ corresponds to a linear arrangement of Zn and C atoms, with the hydrogen atoms of the methyl groups oriented in an eclipsed configuration, resulting in a D_3h_ symmetry point group structure as depicted in Figure 1. The Zn–C and C–H bond lengths in the D_3h_-symmetry global minimum structure of Zn(CH_3_)_2_ are 1.952 and 1.106 Å, respectively. In contrast, the D_3d_-symmetry Zn(CH_3_)_2_ conformer (in which the Zn–C and C–H bond lengths are approximately the same as in the D_3h_-symmetry conformer), with the methyl groups arranged in a staggered configuration (see Figure 1), corresponds to a transition state structure with an a_1u_-symmetry imaginary frequency of 43*i* cm^–1^, associated with the rotation of the methyl groups around the Zn–C bonds.

At this point, it is worth noting that earlier theoretical investigations^24^ predicted the D_3d_-symmetry (i.e., staggered) structure of the Zn(CH_3_)_2_ molecule to be the global minimum. However, these calculations were performed at a much lower theoretical level (i.e., employing the density functional theory method (B3LYP functional) together with a standard Dunning/Huzinaga double-ζ basis set for C and H atoms and a Stuttgart/Dresden effective core potential for Zn), and thus, they should be regarded as considerably less reliable. As demonstrated by our calculations, performed at an advanced *ab initio* level (i.e., using the QCISD method that significantly accounts for electron correlation effects and correlation-consistent basis sets aug-cc-pVDZ and aug-cc-pVTZ), the staggered conformation (D_3d_ symmetry) exhibits saddle point characteristics, while the global minimum of Zn(CH_3_)_2_ molecule corresponds to the eclipsed conformation (D_3h_ symmetry).

**Figure 2:**
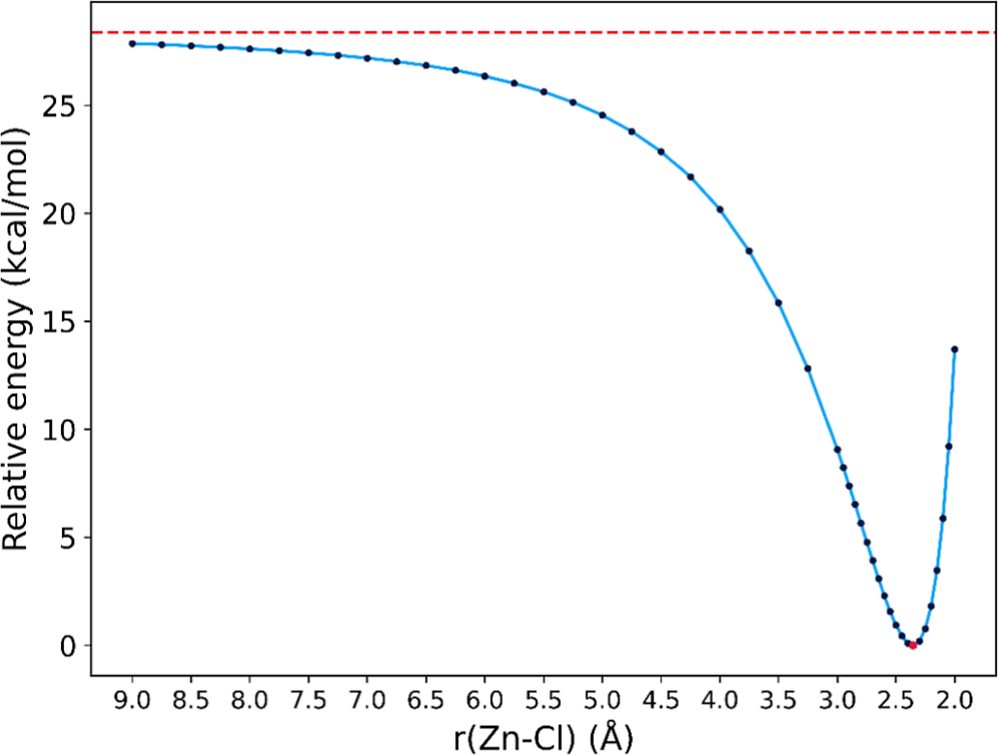
Relative electronic energies (determined at the QCISD/aug-cc-pVDZ theory level) of (CH_3_)_2_Zn···Cl^–^ as a function of the Zn–Cl distance with all other coordinates relaxed to minimize the energy. In red is shown the asymptotic energy of Zn(CH_3_)_2_ + Cl^–^.

Although it may seem that all atoms in the Zn(CH_3_)_2_ molecule are valence-saturated, our calculations indicate that the attachment of a Cl^–^ ion to this molecule is thermodynamically favorable, as suggested by the negative value of Gibbs free energy (Δ*G*_r_^298^) for the process Zn(CH_3_)_2_ + Cl^–^ → (CH_3_)_2_ZnCl^–^ at *T* = 298.15 K, which amounts to −22.88 kcal/mol (the corresponding reaction energy, Δ*E*_r_, is −28.37 kcal/mol). We also verified that the attachment of Cl^–^ to the Zn(CH_3_)_2_ molecule occurs without an energy barrier, as indicated by the energy profile of the reaction (see Figure 2). Indeed, the energy of the entire system decreases as the Cl^–^ ion approaches the dimethylzinc molecule. During this process, the C–Zn–C bond angle decreases from 180° (for isolated, noninteracting Zn(CH_3_)_2_ and Cl^–^) to 138.53° in the equilibrium structure of the (CH_3_)_2_ZnCl^–^ anion (corresponding to a Zn–Cl distance of 2.356 Å, see Figure 2).

**Figure 3:**
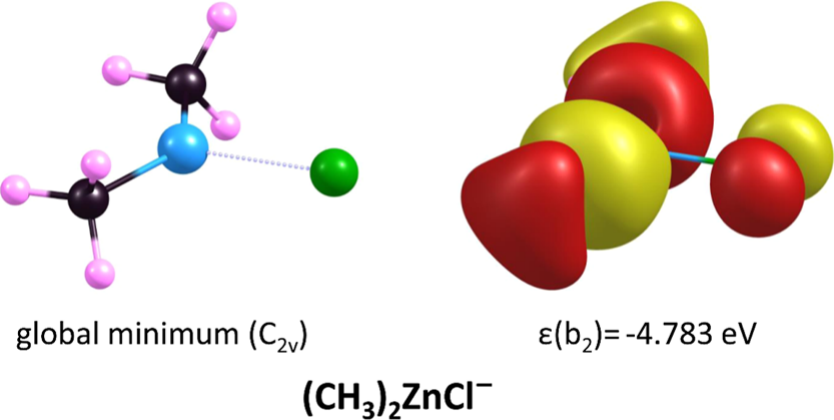
Equilibrium structure of the global minimum of the (CH_3_)_2_ZnCl^–^ anion and its b_2_-symmetry doubly occupied HOMO orbital depicted with a contour spacing of 0.030.

The equilibrium structure of the (CH_3_)_2_ZnCl^–^ anion, formed by the attachment of a Cl^–^ ion to the neutral dimethylzinc molecule, exhibits C_2v_ symmetry (see Figure 3). As mentioned above, the Me–Zn–Me fragment is bent, with a C–Zn–C bond angle of 138.53°, the Zn–C bonds are slightly elongated (by 0.067 Å) compared to the isolated Zn(CH_3_)_2_ molecule, and the Cl atom is attached to Zn atom via a two-electron bond (as confirmed by the predicted σ(Zn–Cl) bond occupancy from NBO analysis approaching 2 electrons) with a Zn–Cl bond length of 2.356 Å. At this point, it is worth noting that the formation of three two-electron bonds by the Zn atom is not surprising, as such systems, containing Zn in the + III oxidation state, have already been reported in the literature.^56,57^

Our discussion of the excess electron binding energy in the (CH_3_)_2_ZnCl^–^ anion necessitates addressing the related issue of excess electron density distribution. Since the anion in question is a closed-shell system, we cannot directly refer to unpaired spin density or to the localization of the “most loosely bound electron”. Within the framework of a single-determinant reference function, we can only consider the two paired electrons described by the highest occupied molecular orbital (HOMO). Unfortunately, the analysis of the HOMO, presented in Figure 3, only confirms the absence of destabilizing antibonding interactions between the ligands (i.e., the two methyl groups and Cl) and the central Zn atom. It does not, however, provide a clear picture of the excess electron density distribution. As a result, within the Hartree–Fock framework, a conclusive analysis of the distribution of the excess electron in the studied anion remains unattainable. To address this limitation, we refer to correlated electron density distributions (calculated at the QCISD level), from which we derived NBO partial atomic charges. According to the NBO population analysis, the excess electron density is distributed between the Cl atom and both methyl groups, which is consistent with the HOMO orbital presented in Figure 3. Specifically, the partial atomic charge on the chlorine atom is −0.722|e|, indicating that we are not dealing with a Zn(CH_3_)_2_···Cl^–^ complex, but rather a [(CH_3_)_2_ZnCl]^−^ system, in which the excess electron density is distributed across the entire molecular framework.

**Figure 4:**
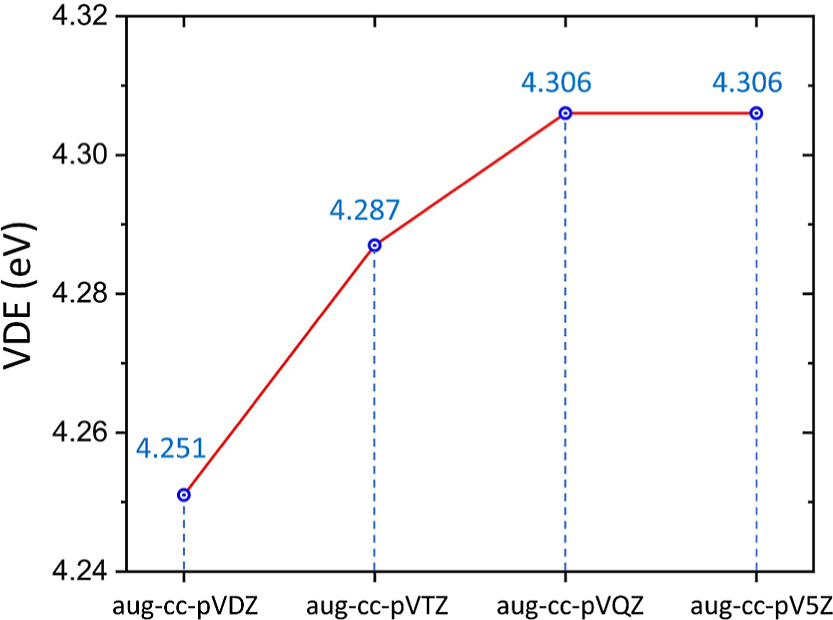
Vertical electron detachment energies (VDE in eV) of the (CH_3_)_2_ZnCl^–^ anion determined with the OVGF method and aug-cc-pVnZ (*n* = D, T, Q, 5) basis sets.

The excess electron is strongly bound within the (CH_3_)_2_ZnCl^–^ anion, as indicated by the calculated vertical electron detachment energy (VDE) of 4.306 eV. Since this value was obtained using the reliable OVGF method and converged with respect to the basis set (see Figure 4), we are confident in its high accuracy.

The (CH_3_)_2_ZnCl^–^ anion is not prone to fragmentation processes, as evidenced by the positive Gibbs free energy values obtained for the reactions (CH_3_)_2_ZnCl^–^ → Zn(CH_3_)_2_ + Cl^–^ (Δ*G*_r_^298^ = 22.88 kcal/mol) and (CH_3_)_2_ZnCl^–^ → C_2_H_6_ (ethane) + ZnCl^–^ (Δ*G*_r_^298^ = 2.62 kcal/mol). Considering all the above, we conclude that the (CH_3_)_2_ZnCl^–^ anion is both electronically and thermodynamically stable, and that its formation via the attachment of a Cl^–^ ion to the dimethylzinc molecule should proceed spontaneously, without a kinetic barrier. Thus, it may serve as an intermediate compound in the initial stage of the dimethylzinc stabilization process.

Having discussed the molecular-level process of the formation of the (CH_3_)_2_ZnCl^–^ anion through the attachment of a Cl^–^ ion to the dimethylzinc molecule, as well as the electronic and thermodynamic stability of this anion, we now move on to proposing experimental pathways for generating the (CH_3_)_2_ZnCl^–^ anion. The first of the proposed reactions is the most straightforward approach, involving the direct nucleophilic addition of a halide ion to dimethylzinc using a halide salt, such as tetrabutylammonium chloride.^57,58^ This reaction is likely to be carried out in a polar aprotic solvent, such as tetrahydrofuran (THF) or diethyl ether, which can dissolve both the quaternary ammonium salt and dimethylzinc without directly reacting with the dimethylzinc itself. The large quaternary ammonium cation (NBu_4_^+^) is expected to ensure the solubility of the halide in organic solvents like THF or ether, allowing the halide ion to nucleophilically attack the zinc center. The second proposed pathway involves the reaction with a suitable halide-containing organometallic reagent. Since dimethylzinc is suggested to be able to react with organometallic compounds containing halide anions, potentially leading to the transfer of a halide ion to the zinc center,^6^ the reaction of Zn(CH_3_)_2_ with a proper Grignard reagent (i.e., methylmagnesium chloride (CH_3_MgCl), which contains both a methyl group and a halide anion) could be explored. This reaction is expected to result in the formation of (CH_3_)_2_ZnCl^–^ and CH_3_Mg^+^. Similar to the previous procedure, a suitable solvent for this pathway appears to be diethyl ether or THF.

### 3.3 Formation of the (CH_3_)_2_ZnClLi and (CH_3_)_2_ZnClNa Salts

The second stage in the process of stabilizing dimethylzinc involves the formation of a salt through the neutralization of the (CH_3_)_2_ZnCl^–^ anion by attaching a Li^+^ or Na^+^ cation. As expected, the addition of a lithium or sodium cation to the (CH_3_)_2_ZnCl^–^ system is highly energetically favorable, as it leads to charge neutralization. Indeed, our calculations revealed that the Gibbs free energy values at *T* = 298.15 K for the reactions (CH_3_)_2_ZnCl^–^ + Li^+^ → (CH_3_)_2_ZnClLi and (CH_3_)_2_ZnCl^–^ + Na^+^ → (CH_3_)_2_ZnClNa are negative, amounting to −131.92 and −108.90 kcal/mol, respectively (the corresponding Δ*E*_r_ values are −141.49 and −117.80 kcal/mol). Due to the nature of these processes, which involve charge neutralization through the combination of oppositely charged ions, no kinetic barriers are expected during their occurrence.

**Figure 5:**
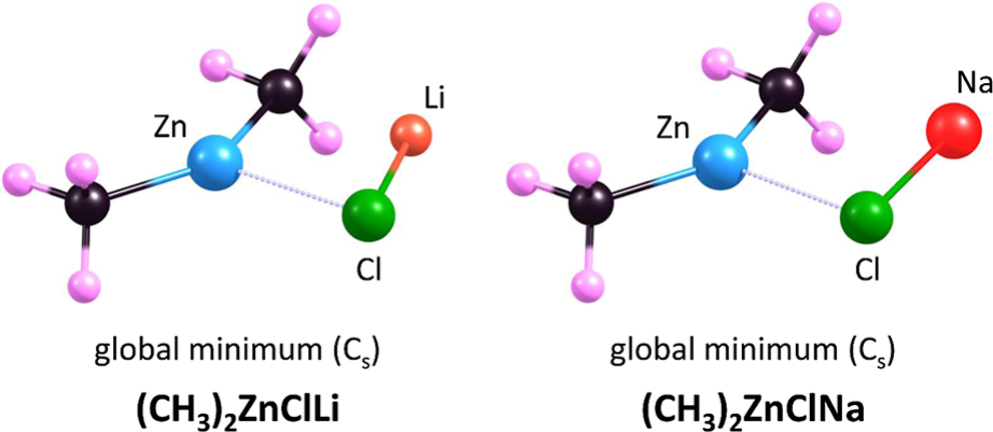
Equilibrium structures of the global minimum of the (CH_3_)_2_ZnClLi and (CH_3_)_2_ZnClNa salts.

The equilibrium structures of the (CH_3_)_2_ZnClLi and (CH_3_)_2_ZnClNa salts are shown in Figure 5. Both molecules exhibit *C*_s_ symmetry, with a symmetry plane including the C, Zn, Cl, and Li/Na atoms, as well as two of the six H atoms. The C–Zn–C bond angles in the (CH_3_)_2_ZnClLi and (CH_3_)_2_ZnClNa molecules are 145.94° and 143.71°, respectively, which are similar to the angle determined for the (CH_3_)_2_ZnCl^–^ anion (138.53°), albeit slightly larger (by about 6–8°). The Zn–C bond lengths in the lithium and sodium salts are 2.059 and 1.976 Å, respectively, and are close to the corresponding values for the (CH_3_)_2_ZnCl^–^ anion (2.020 Å) and Zn(CH_3_)_2_ (1.952 Å) (see the preceding section). Our calculations showed that salt formation leads to an elongation of the Zn–Cl bond (compared to the anion), specifically by 0.124 Å in (CH_3_)_2_ZnClLi and 0.103 Å in (CH_3_)_2_ZnClNa, resulting in Zn–Cl bond lengths of 2.480 and 2.459 Å, respectively.

The (CH_3_)_2_ZnClLi and (CH_3_)_2_ZnClNa salts are polar, as evidenced by their dipole moments (calculated using QCISD electron density) of 5.808 and 8.567 D, respectively. Unsurprisingly, the directions of the dipole moment vectors in both salts are approximately aligned with the Li(Na)–Cl bond directions. Since the Li–Cl bond length in (CH_3_)_2_ZnClLi (2.169 Å) is only slightly longer (by 0.084 Å) than that in the isolated LiCl molecule (calculated at the same theory level), and the Na–Cl bond length in (CH_3_)_2_ZnClNa (2.519 Å) is only slightly longer (by 0.094 Å) than that in the isolated NaCl molecule (also calculated at the same theory level), and considering that NBO population analysis predicts partial atomic charges of +0.926|e| and −0.720|e| for Li and Cl in (CH_3_)_2_ZnClLi, and +0.963|e| and −0.744|e| for Na and Cl in (CH_3_)_2_ZnClNa, the (CH_3_)_2_ZnClLi and (CH_3_)_2_ZnClNa salts can be viewed as systems composed of strongly interacting dimethylzinc and alkali metal chloride units. Our calculations further revealed that both (CH_3_)_2_ZnClLi and (CH_3_)_2_ZnClNa salts are thermodynamically stable at room temperature, indicating that they are not prone to any fragmentation processes. Specifically, we found that the Gibbs free energy values for the reactions (CH_3_)_2_ZnClLi → Zn(CH_3_)_2_ + LiCl and (CH_3_)_2_ZnClNa → Zn(CH_3_)_2_ + NaCl are positive, amounting to 10.99 and 9.19 kcal/mol, respectively. The observed stability of these salts is promising for their potential application as stable systems containing dimethylzinc.

Having discussed the molecular-level processes involved in the formation of the (CH_3_)_2_ZnClLi and (CH_3_)_2_ZnClNa salts through the attachment of Li^+^ or Na^+^ ions to the dimethylzinc molecule, as well as the structural and thermodynamic stability of these compounds, we now turn to proposing experimental pathways^59−62^ for generating the (CH_3_)_2_ZnClLi and (CH_3_)_2_ZnClNa salts. To neutralize dimethylzinc chloride (CH_3_)_2_ZnCl^–^ anions with lithium (Li^+^) or sodium (Na^+^) cations and form stable salts (i.e (CH_3_)_2_ZnClLi or (CH_3_)_2_ZnClNa), several practical aspects must be considered, such as reagent solubility, the stability of the resulting salts, and the inertness of the solvent.

It appears that lithium or sodium cations can be introduced using soluble salts like lithium chloride (LiCl) or sodium chloride (NaCl) in a nonreactive, polar aprotic solvent such as THF, diethyl ether, or acetonitrile. We propose the use of these polar aprotic solvents, as they are inert toward organozinc compounds (and thus do not interfere with the reaction) and are expected to dissolve both the dimethylzinc anionic complex and the lithium or sodium salt. Once (CH_3_)_2_ZnCl^–^ is dissolved in an appropriate solvent, a lithium or sodium salt (such as LiCl, NaCl, or another soluble salt like LiBF_4_ or NaPF_6_) should be slowly added to the solution, enabling the cation (Li^+^ or Na^+^) to pair with the (CH_3_)_2_ZnCl^–^ anion and thus forming the desired salt.^57,62^

It is likely that proper execution of this process may require some mixing and stirring over a specified period to ensure complete ion pairing. However, the reaction should proceed spontaneously, driven by the thermodynamic stability of the resulting salt. The final isolation of the salt may involve solvent evaporation under reduced pressure, followed by recrystallization from a suitable solvent for purification. We believe this procedure should yield the desired neutral lithium or sodium salt of dimethylzinc chloride in a straightforward and thermodynamically favorable manner.

### 3.4 Recovery of Dimethylzinc from (CH_3_)_2_ZnClLi and (CH_3_)_2_ZnClNa Salts

As explained in the preceding section, both (CH_3_)_2_ZnClLi and (CH_3_)_2_ZnClNa salts are thermodynamically stable at room temperature, meaning they are not susceptible to fragmentation processes. While this stability is beneficial for the intended goal of stabilizing dimethylzinc, it poses a challenge when it comes to the recovery of dimethylzinc from these salts. However, the fact that the Gibbs free energy values for the fragmentation reactions yielding Zn(CH_3_)_2_ and either LiCl or NaCl, although positive (10.99 and 9.19 kcal/mol, respectively), are not excessively high, suggests that the detachment of sodium or lithium chloride from the (CH_3_)_2_ZnClLi and (CH_3_)_2_ZnClNa salts can likely be induced without significant difficulty. Unfortunately, theoretical calculations cannot provide sufficient guidance for proposing experimental routes for the recovery of dimethylzinc, and thus this section, by necessity, will be the most speculative of all. Here, we will cautiously propose several potential procedures, hoping that one will prove effective.

Therefore, knowing that both (CH_3_)_2_ZnLiCl and (CH_3_)_2_ZnNaCl salts are stable, and that the thermodynamic barrier for detaching LiCl or NaCl from them is approximately 9–11 kcal/mol, we suggest several strategies for promoting this detachment.^63^ The first strategy involves the use of polar aprotic solvents, such as dimethyl sulfoxide (DMSO), dimethylformamide (DMF), or acetonitrile (MeCN), which can solvate the cations (Li^+^ or Na^+^) and promote dissociation of the ion pairs.^64,65^ The polarity of these solvents may destabilize the salts by efficiently solvating the metal cations, thus weakening the interaction between the metal and the chloride ion. The second strategy involves chelation with crown ethers or cryptands, which can selectively bind the metal cations (Li^+^ or Na^+^), effectively removing them from the ion pair and facilitating chloride dissociation.^66−70^ For example, 15-crown-5 and 12-crown-4 are capable of chelating Na^+^ and Li^+^, respectively, while certain cryptands (such as crypt-222) are even more effective, fully encapsulating the cations. Since strong binding of the cation by the chelator disrupts the ionic bond between the cation and chloride, adding crown ethers or cryptands to the salt solution should drive the desired dissociation. A third strategy could involve thermal dissociation, where gradually heating the salt would provide the necessary energy to overcome the thermodynamic barrier of approximately 9–11 kcal/mol, leading to dissociation into free ions.^55,71^ The fourth approach could utilize halide scavenging with Lewis acids. Namely, adding a strong Lewis acid, such as aluminum chloride (AlCl_3_) or boron trifluoride (BF_3_), can act as a halide scavenger by binding to the chloride ion and pulling it away from the metal cation, promoting salt dissociation (Lewis acids have a high affinity for halide ions, particularly chloride, which shifts the equilibrium in favor of dissociation).^56,59,72,73^ Another approach involves the use of ionic liquids or molten salt systems, which provide a highly dissociative medium for the salts. These media are known for stabilizing ions in a dissociated state and promoting the separation of ion pairs. Given their capacity to facilitate free movement of ions, their application may lead to easier dissociation of LiCl or NaCl from the complex.^74−76^ Finally, electrochemical dissociation could be employed, as applying an electrical potential in an electrochemical cell may induce dissociation of the salt by driving ion migration. The appropriate voltage is expected to separate the cation (Li^+^ or Na^+^) from the chloride ion by promoting their movement to opposite electrodes.^74−78^

## 4 Summary

On the basis of the ab initio electronic structure calculations carried out using the QCISD and OVGF methods with the aug-cc-pV*n*Z (*n* = D, T, Q, 5) basis sets performed for the neutral dimethylzinc molecule (Zn(CH_3_)_2_), the (CH_3_)_2_ZnCl^–^ anion, and the (CH_3_)_2_ZnClLi and (CH_3_)_2_ZnClNa salts, we arrive at the following conclusions:

(i) The reactive dimethylzinc molecule can be stabilized through a two-step process: first, by attaching a Cl^–^ ion to Zn(CH_3_)_2_ to form the (CH_3_)_2_ZnCl^–^ anion, followed by the attachment of either a Li^+^ or Na^+^ cation to yield the (CH_3_)_2_ZnClLi or (CH_3_)_2_ZnClNa salt. Both of these processes are thermodynamically favorable at room temperature and proceed without kinetic barriers.
(ii) The (CH_3_)_2_ZnCl^–^ anion, which plays a key role in stabilizing dimethylzinc, is both electronically and thermodynamically stable, with a vertical electron detachment energy of 4.306 eV.
(iii) The final products of dimethylzinc stabilization, the (CH_3_)_2_ZnClLi and (CH_3_)_2_ZnClNa salts, are ionic in nature, thermodynamically stable, and should not undergo any fragmentation processes at room temperature.
(iv) Dimethylzinc molecules can be recovered from the (CH_3_)_2_ZnClLi and (CH_3_)_2_ZnClNa salts through suggested processes such as thermal dissociation, dissociation of ion pairs promoted by polar aprotic solvents or ionic liquids, chelation with crown ethers or cryptands, halide scavenging with Lewis acids, or electrochemical dissociation.

## References

[ref1] Seyferth D. Zinc Alkyls, Edward Frankland, and the Beginnings of Main-Group Organometallic Chemistry. Organometallics 2001, 20, 2940–2955. 10.1021/om010439f.

[ref2] Frankland E. On the isolation of the organic radicals. Q. J. Indian Chem. Soc. 1850, 2, 263–296. 10.1039/QJ8500200263.

[ref3] Ender E. Organozinc Reagents in Organic Synthesis; CRC Press, 1996.

[ref4] Grévy J. Zinc: Organometallic Chemistry in Encyclopedia of Inorganic Chemistry; Wiley, 2006.

[ref5] Boersma J. Comprehensive Organometallic Chemistry; Pergamon Press, 1982, p 823.

[ref6] Knochel P.; Singer R. D. Preparation and reactions of polyfunctional organozinc reagents in organic synthesis. Chem. Rev. 1993, 93, 2117–2188. 10.1021/cr00022a008.

[ref7] Knochel P. Stereoselective Reactions Mediated by Functionalized Diorganozincs. Synlett 1995, 1995, 393–403. 10.1055/s-1995-4997.

[ref8] Nakamura M.; Nakamura E. Preparative Routes to Organozinc Reagents Used for Organic Synthesis. J. Synth. Org. Chem. 1998, 56, 632–644. 10.5059/yukigoseikyokaishi.56.632.

[ref9] Knochel P.; Almena Perea J. J.; Jones P. Organozinc mediated reactions. Tetrahedron 1998, 54, 8275–8319. 10.1016/S0040-4020(98)00318-4.

[ref10] Boudier A.; Bromm L. O.; Lotz M.; Knochel P. New Applications of Polyfunctional Organometallic Compounds in Organic Synthesis. Angew. Chem., Int. Ed. 2000, 39, 4414–4435. 10.1002/1521-3773(20001215)39:24<4414::AID-ANIE4414>3.0.CO;2-C. 11169635

[ref11] Pierson H. O., Handbook of Chemical Vapor Deposition Principles, Technology and Applications, 2nd ed., Elsevier, 1999.

[ref12] Weiss F.; Audier M.; Bartasyte A.; Bellet D.; Girardot C.; Jimenez C.; Kreisel J.; Pignard S.; Salaun M.; Ternon C. Multifunctional oxide nanostructures by metal-organic chemical vapor deposition (MOCVD). Pure Appl. Chem. 2009, 81, 1523–1534. 10.1351/PAC-CON-08-08-10.

[ref13] Stringfellow G. B. Organometallic Vapor-Phase Epitaxy: Theory and Practice; Academic Press, Inc.: San Diego, 1989.

[ref14] Jones A. C.; Hitchman M. L. Chemical Vapor Deposition: Precursors, Processes and Applications; Royal Society of Chemistry Publishing: London, 2009.

[ref15] Afzaal M.; Malik M. A.; O’Brien P. Preparation of zinc containing materials. New J. Chem. 2007, 31, 2029–2040. 10.1039/b712235g.

[ref16] Song Y.; Mendes P. C. D.; Kozlov S. M. Tunable properties and composition of ZnO films supported on metal surfaces, Tunable properties and composition of ZnO films supported on metal surfaces. J. Mater. Chem. A 2023, 11, 13665–13676. 10.1039/D3TA01940C.

[ref17] Lo Presti F.; Pellegrino A. L.; Malandrino G. Metal-Organic Chemical Vapor Deposition of Oxide Perovskite Films: A Facile Route to Complex Functional Systems. Adv. Mater. Interfaces 2022, 9, 2102501 10.1002/admi.202102501.

[ref18] Wang J.; Isshiki M. Handbook of Electronic and Photonic Materials; Springer: New York, 2006.

[ref19] Abedsoltan H.; Shiflett M. B. Mitigation of Potential Risks in Chemical Laboratories: A Focused Review. ACS Chem. Health Saf. 2024, 31 (2), 104–120. 10.1021/acs.chas.3c00097.

[ref20] Ashraf S.; Jones A. C.; Bacsa J.; Steiner A.; Chalker P. R.; Beahan P.; Hindley S.; Odedra R.; Williams P. A.; Heys P. N. MOCVD of Vertically Aligned ZnO Nanowires Using Bidentate Ether Adducts of Dimethylzinc. Chem. Vap. Deposition 2011, 17, 45–53. 10.1002/cvde.201006881.

[ref21] Van Der Kerk G. J. M. Organozinc coordination chemistry and catalytic effects of organozinc coordination compounds. Pure Appl. Chem. 1972, 30 (3–4), 389–408. 10.1351/pac197230030389.

[ref22] Pajerski A. D.; Bergstresser G. L.; Parvez M.; Richey H. G. Jr. X-ray structures of threaded diethylmagnesium(18-crown-6) and diethylzinc(18-crown-6). J. Am. Chem. Soc. 1988, 110, 4844–4845. 10.1021/ja00222a062.

[ref23] O’Brien P.; Hursthouse M. B.; Motevalli M.; Walsh J. R.; Jones A. C. Structural studies of some Group 12 metal alkyl adducts: the X-ray crystal structures of Me_2_Zn[Me_2_N(CH_2_) _2_NMe_2_], Me_2_Cd[Me_2_N(CH_2_) _2_NMe_2_], (Me_3_CCH_2_)_2_ Zn[Me_2_N(CH_2_) _2_NMe_2_] and (Me_3_CCH_2_) _2_Cd[Me_2_N(CH_2_)_2_ NMe_2_]. J. Organomet. Chem. 1993, 449, 1–8. 10.1016/0022-328X(93)80100-P.

[ref24] Haaland A.; Green J. C.; McGrady G. S.; Downs A. J.; Gullo E.; Lyall M. J.; Timberlake J.; Tutukin A. V.; Volden H. V.; Østby K.-A. The length, strength and polarity of metal–carbon bonds: dialkylzinc compounds studied by density functional theory calculations, gas electron diffraction and photoelectron spectroscopy. Dalton Trans. 2003, 4356–4366. 10.1039/B306840B.

[ref25] Antes I.; Frenking G. Theoretical Studies of Organometallic Compounds. XIV. Structure and Bonding of the Transition Metal Methyl and Phenyl Compounds MCH3 and MC6H5 (M = Cu, Ag, Au) and M(CH3)2 and M(C6H5)2 (M = Zn, Cd, Hg). Organometallics 1995, 14, 4263–4268. 10.1021/om00009a032.

[ref26] Knight D. A.; Zidan R.; Lascola R.; Mohtadi R.; Ling C.; Sivasubramanian P.; Kaduk J. A.; Hwang S.-J.; Samanta D.; Jena P. Synthesis, Characterization, and Atomistic Modeling of Stabilized Highly Pyrophoric Al(BH_4_)_3_ Via the Formation of the Hypersalt K[Al(BH_4_)_4_]. J. Phys. Chem. C 2013, 117 (39), 19905–19915. 10.1021/jp407230a.

[ref27] Pople J. A.; Head-Gordon M.; Raghavachari K. Quadratic configuration interaction. A general technique for determining electron correlation energies. J. Chem. Phys. 1987, 87, 5968–5975. 10.1063/1.453520.

[ref28] Gauss J.; Cremer D. Analytical evaluation of energy gradients in quadratic configuration interaction theory. Chem. Phys. Lett. 1988, 150, 280–286. 10.1016/0009-2614(88)80042-3.

[ref29] Salter E. A.; Trucks G. W.; Bartlett R. J. Analytic energy derivatives in many-body methods. I. First derivatives. J. Chem. Phys. 1989, 90, 1752–1766. 10.1063/1.456069.

[ref30] Dunning T. H. Jr. Gaussian basis sets for use in correlated molecular calculations. I. The atoms boron through neon and hydrogen. J. Chem. Phys. 1989, 90, 1007–1023. 10.1063/1.456153.

[ref31] Kendall R. A.; Dunning T. H. Jr.; Harrison R. J. Electron affinities of the first-row atoms revisited. Systematic basis sets and wave functions. J. Chem. Phys. 1992, 96, 6796–6802. 10.1063/1.462569.

[ref32] Cížek J.; Advances in Chemical Physics; Wiley Interscience, 1969.

[ref33] Purvis G. D. III; Bartlett R. J. A full coupled-cluster singles and doubles model – the inclusion of disconnected triples. J. Chem. Phys. 1982, 76, 1910–1918. 10.1063/1.443164.

[ref34] Scuseria G. E.; Janssen C. L.; Schaefer H. F. III An efficient reformulation of the closed-shell coupled cluster single and double excitation (CCSD) equations. J. Chem. Phys. 1988, 89, 7382–7387. 10.1063/1.455269.

[ref35] Bartlett R. J.; Purvis G. D. III Many-body perturbation theory, coupled-pair many-electron theory, and the importance of quadruple excitations for the correlation problem. Int. J. Quantum Chem. 1978, 14, 561–581. 10.1002/qua.560140504.

[ref36] Zakrzewski V. G.; Ortiz J. V.; Nichols J. A.; Heryadi D.; Yeager D. L.; Golab J. T. Comparison of perturbative and multiconfigurational electron propagator methods. Int. J. Quantum Chem. 1996, 60, 29–36. 10.1002/(SICI)1097-461X(1996)60:1<29::AID-QUA3>3.0.CO;2-7.

[ref37] Simons J. Direct Calculation of First- and Second-Order Density Matrices. The Higher RPA Method. J. Chem. Phys. 1971, 55, 1218–1230. 10.1063/1.1676208.

[ref38] Ortiz J. V. Electron binding energies of anionic alkali metal atoms from partial fourth order electron propagator theory calculations. J. Chem. Phys. 1988, 89, 6348–6352. 10.1063/1.455401.

[ref39] Rowe D. J. Equations-of-Motion Method and the Extended Shell Model. Rev. Mod. Phys. 1968, 40, 153–166. 10.1103/RevModPhys.40.153.

[ref40] Cederbaum L. S. One-body Green’s function for atoms and molecules: theory and application. J. Phys. B: At., Mol. Opt. Phys. 1975, 8, 290–303. 10.1088/0022-3700/8/2/018.

[ref41] Simons J. Energy-Shift Theory of Low-Lying Excited Electronic States of Molecules. J. Chem. Phys. 1972, 57, 3787–3792. 10.1063/1.1678845.

[ref42] Simons J.; Smith W. D. Theory of electron affinities of small molecules. J. Chem. Phys. 1973, 58, 4899–4907. 10.1063/1.1679074.

[ref43] Zakrzewski V. G.; Ortiz J. V. Semidirect algorithms for third-order electron propagator calculations. Int. J. Quantum Chem. 1995, 53, 583–590. 10.1002/qua.560530602.

[ref44] Zakrzewski V. G.; Ortiz J. V. Semidirect algorithms in electron propagator calculations. Int. J. Quantum Chem. 1994, 52, 23–27. 10.1002/qua.560520806.

[ref45] Woon D. E.; Dunning T. H. Jr. Gaussian basis sets for use in correlated molecular calculations. III. The atoms aluminum through argon. J. Chem. Phys. 1993, 98, 1358–1371. 10.1063/1.464303.

[ref46] Peterson K. A.; Woon D. E.; Dunning T. H. Jr. Benchmark calculations with correlated molecular wave functions. IV. The classical barrier height of the H+H2→H2+H reaction. J. Chem. Phys. 1994, 100, 7410–7415. 10.1063/1.466884.

[ref47] Zakrzewski V. G.; Dolgounitcheva O.; Ortiz J. V. Ionization energies of anthracene, phenanthrene, and naphthacene. J. Chem. Phys. 1996, 105, 8748–8753. 10.1063/1.472654.

[ref48] Foster J. P.; Weinhold F. Natural hybrid orbitals. J. Am. Chem. Soc. 1980, 102, 7211–7218. 10.1021/ja00544a007.

[ref49] Reed A. E.; Weinhold F. Natural bond orbital analysis of near-Hartree–Fock water dimer. J. Chem. Phys. 1983, 78, 4066–4073. 10.1063/1.445134.

[ref50] Reed A. E.; Weinstock R. B.; Weinhold F. Natural population analysis. J. Chem. Phys. 1985, 83, 735–746. 10.1063/1.449486.

[ref51] Carpenter J. E.; Weinhold F. Analysis of the geometry of the hydroxymethyl radical by the “different hybrids for different spins” natural bond orbital procedure. J. Mol. Struct. 1988, 169, 41–62. 10.1016/0166-1280(88)80248-3.

[ref52] Reed A. E.; Curtiss L. A.; Weinhold F. Intermolecular interactions from a natural bond orbital, donor-acceptor viewpoint. Chem. Rev. 1988, 88, 899–926. 10.1021/cr00088a005.

[ref53] NBO 7.0; Glendening E. D.; Badenhoop J. K.; Reed A. E.; Carpenter J. E.; Bohmann J. A.; Morales C. M.; Karafiloglou P.; Landis C. R.; Weinhold F. Theoretical Chemistry Institute; University of Wisconsin: Madison, WI, 2018.

[ref54] Frisch M. J.; Trucks G. W.; Schlegel H. B.; Scuseria G. E.; Robb M. A.; Cheeseman J. R.; Scalmani G.; Barone V.; Petersson G. A.; Nakatsuji H.; Li X.; Gaussian 16, Revision C.01; Gaussian, Inc.: Wallingford CT, 2016.

[ref55] Samanta D.; Jena P. Zn in the + III Oxidation State. J. Am. Chem. Soc. 2012, 134, 8400–8403. 10.1021/ja3029119. 22559713

[ref56] Fang H.; Banjade H.; Deepika D.; Jena P. Realization of the Zn3+ Oxidation State. Nanoscale 2021, 13, 14041–14048. 10.1039/D1NR02816B. 34477685

[ref57] Hartwig J. F. Organotransition Metal Chemistry: From Bonding to Catalysis; University Science Books, 2010.

[ref58] Rappoport Z.; Marek I. The Chemistry of Organozinc Compounds: R–Zn, w Patai Series: The Chemistry of Functional Groups; Wiley, 2006.

[ref59] Knochel P. Organometallic Complexes of Zinc. In Science of Synthesis, Category 1: Organometallic; O’Neil I. A., Ed.; Georg Thieme Verlag KG, 2004.

[ref60] Zhang B.; Li S.; Cokoja M.; Herdtweck E.; Mink J.; Zang S.-L.; Herrmann W. A.; Kühn F. E. Ion Pairs of Weakly Coordinating Cations and Anions: Synthesis and Application for Sulfide to Sulfoxide Oxidations. Z. Naturforsch. B 2014, 69 (11–12), 1149–1163. 10.5560/znb.2014-4165.

[ref61] Balkenhohl M.; Knochel P. Recent Advances of the Halogen–Zinc Exchange Reaction. Chem. - Eur. J. 2020, 26 (17), 3688–3697. 10.1002/chem.201904794. 31742792 PMC7155102

[ref62] Knochel P.; Jones P. Organozinc Reagents; Oxford University Press, 1999.

[ref63] Dyagileva L. M.; Aleksandrova Yu. A. The Reactivity of Organozinc and Organocadmium Compounds in Decomposition Reactions. Russ. Chem. Rev. 1986, 55, 1054–1061. 10.1070/RC1986v055n11ABEH003240.

[ref64] Reichardt C. Solvents and Solvent Effects in Organic Chemistry, 3rd ed., Wiley, 2003.

[ref65] Marcus Y. Ion Solvation; Wiley-Interscience, 1985.

[ref66] Cram D. J.; Cram J. M. Host-Guest Chemistry: Complexes between organic compounds simulate the substrate selectivity of enzymes. Science 1974, 183, 803–809. 10.1126/science.183.4127.803. 17780761

[ref67] Cram D. J.; Helgeson R. C.; Sousa L. R.; Timko J. M.; Newcomb M.; Moreau P.; de Jong F.; Gokel G. W.; Hoffman D. H.; Domeier L. A.; et al. Chiral recognition in complexation of guests by designed host molecules. Pure Appl. Chem. 1975, 43, 327–349. 10.1351/pac197543030327.

[ref68] Lehn J.-M. Supramolecular Chemistry: Concepts and Perspectives; Wiley, 1995.

[ref69] Goldberg I. Complexes of Crown Ethers with Molecular Guests; Academic Press: London, 1984, pp 261–335.

[ref70] Gokel G. W. Crown Ethers and Cryptands; John Wiley & Sons Ltd., 1996, pp 263–307.

[ref71] Shriver D. F.; Drezdzon M. A. The Manipulation of Air-Sensitive Compounds; Wiley-Interscience, 1986.

[ref72] Sun J.; Wang L.; Zhang S.; Li Z.; Zhang X.; Dai W.; Mori R. ZnCl2/phosphonium halide: An efficient Lewis acid/base catalyst for the synthesis of cyclic carbonate. J. Mol. Catal. A: Chem. 2006, 256, 295–300. 10.1016/j.molcata.2006.05.004.

[ref73] Koop S.; Mrózek O.; Janiak L.; Belyaev A.; Putscher M.; Marian C. M.; Steffen A. Synthesis, Structural Characterization, and Phosphorescence Properties of Trigonal Zn(II) Carbene Complexes. Inorg. Chem. 2024, 63, 891–901. 10.1021/acs.inorgchem.3c03915. 38118184

[ref74] Mallakpour S.; Dinari M. Ionic Liquids as Green Solvents: Progress and Prospects. In Green Solvents II; Mohammad A., Inamuddin D., Eds.; Springer, 2012.

[ref75] Rogers R. D.; Seddon K. R. Ionic Liquids as Green Solvents: Progress and Prospects; American Chemical Society, 2003.

[ref76] Ohno H. Electrochemical Aspects of Ionic Liquids; Wiley-Interscience, 2005.

[ref77] Gritzner G.; Kuta J. Recommendations on reporting electrode potentials in nonaqueous solvents (Recommendations 1983). Pure Appl. Chem. 1984, 56, 461–466. 10.1351/pac198456040461.

[ref78] Bard A. J.; Faulkner L. R. Electrochemical Methods: Fundamentals and Applications, 2nd ed., Wiley, 2000.

